# AI-Driven Patient Screening for Clinical Trials in Pancreatic Cancer: The PANCR-AI Pilot Retrospective Comparative Study

**DOI:** 10.2196/80268

**Published:** 2026-02-23

**Authors:** Arthur Claessens, Alizée Simon, Agathe Manchart, Laura Desmonts, Cassandre Leguay, Jean-Christophe Faivre, Sébastien Cambier, Julia Salleron, Aurélien Lambert

**Affiliations:** 1 Université de Lorraine, Nancy, France; 2 Medical Oncology Department, Institut de Cancérologie de Lorraine, 6 Avenue de Bourgogne, Vandoeuvre-Lès-Nancy, 54519, France, 33 383-598-400; 3 Radiotherapy Department, Institut de Cancérologie de Lorraine, Vandoeuvre-Lès-Nancy, France; 4 Biostatistics Department, Institut de Cancérologie de Lorraine, Vandoeuvre-Lès-Nancy, France; 5 Interdisciplinarité en Santé Publique, Interventions & Instruments de mesure complexes – Région Est, INSPIIRE UMR 1319, INSERM, Université de Lorraine, Nancy, France

**Keywords:** pancreatic neoplasms, artificial intelligence, AI, reproducibility of results, clinical trials as topic, pilot project

## Abstract

**Background:**

Screening for clinical trials is challenging for clinicians due to its time-consuming and repetitive nature. The rise of artificial intelligence (AI) offers an opportunity to improve screening productivity and reproducibility. Pancreatic cancer is characterized by increasing incidence, poor survival outcomes, and an urgent need for improved management strategies.

**Objective:**

This study aimed to assess the performance of AI in evaluating clinical trial inclusion and exclusion criteria, compared to a double-blind human gold standard, using a retrospective cohort.

**Methods:**

In the PANCR-AI (Pancreatic Cancer Retrospective Screening with Artificial Intelligence) pilot study, we retrospectively reviewed cases from our institutional database of patients with advanced pancreatic cancer presented at tumor board meetings between January 2018 and December 2023. Each patient was screened for clinical trials open for inclusion at the time of the multidisciplinary meeting. Manual screening of eligibility criteria for each patient-trial pair was performed by 2 blinded oncologists to determine potential eligibility (gold standard), with a third oncologist resolving discrepancies. Potential eligibility was also assessed using 3 large language models (ie, GPT-4.5, Claude 3.7 Sonnet, and Mistral-7B-Instruct v0.3). Their performance was compared to the human gold standard using standard evaluation metrics (eg, sensitivity, specificity, precision, recall, and *F*_1_-score). Correlations between the risk of failure and the number of words and characters in the criteria were analyzed. The time required to complete the screening was recorded for both human and AI assessments. The number of trials open for enrollment at the time of the tumor board meeting was also recorded as a variable for analysis.

**Results:**

Across 341 patient-trial pairs, the AI models demonstrated high sensitivity, ranging from 83.3% to 92.2%. Analysis of the criteria showed a correlation between the risk of failure and the number of words and the number of characters in the criteria. Overall screening time for manual assessment was significantly longer for the human gold standard (44.70 hours) assessment than for AI (2.53-3.15 hours). Patients were more likely to have been included in a clinical trial if the number of trials open for enrollment was higher at the time of the tumor board meeting (*P*=.02).

**Conclusions:**

Our study highlights the promising performance of AI in clinical trial screening. Future work should explore integration with structured clinical data, such as laboratory values or radiological findings, to improve multimodal comprehension. Expanding the evaluation to a broader range of tumor types and multicenter datasets would improve generalizability. Finally, real-time prospective validation and workflow integration with electronic health records will be critical to assess the feasibility and clinical impact of large language model–assisted screening in daily oncology practice. Addressing these challenges will be essential to move from proof of concept to scalable clinical implementation.

## Introduction

Pancreatic cancer is a leading cause of cancer-related mortality, and its incidence continues to rise [1]. In advanced and metastatic cancer, therapeutic options are often limited, with poor results for overall survival in comparison with other malignancies [2,3]. There is a critical need to gather knowledge about the different subtypes of the disease and to adopt new approaches to improve patient outcomes. Clinical trials are the preferred option to ensure access to innovation. Screening patients for clinical trials is a complex and time-consuming procedure. Screening electronic patient records requires reviewing all inclusion and exclusion criteria for each potential clinical trial for which a patient might be eligible. This effort becomes obsolete as soon as new elements are added to the patient medical record. This assessment at a given moment does not predict short- or medium-term clinical evolution, particularly in the case of patients with locally advanced and metastatic pancreatic adenocarcinomas. The multidisciplinary tumor board meeting is held at a critical moment in the therapeutic decision-making process and represents an opportunity to suggest the inclusion of a patient in a clinical trial before treatment begins.

Promising artificial intelligence (AI) tools, such as assistant agents, are increasingly being incorporated into hospital information systems, and their applications now cover a wide range of needs expressed by health care professionals [4]. The emergence of generative AI could help physicians to screen patients by performing automated, real-time analysis of their electronic medical records to determine whether the inclusion and exclusion criteria for a clinical trial are met. This real-time screening would increase the chances of offering the patient a relevant clinical trial.

Screening for clinical trials at the time of the pretherapeutic tumor board meetings is of major interest for the prognosis of pancreatic malignancies. Current efforts in this area are hampered by a lack of human resources. Among the possible factors leading to the failure of clinical trials, the small number of patients included is one of the main causes of trial discontinuation [5]. Only an estimated 2% to 4% of all patients with cancer participate in clinical trials. Although multiple factors explain this low enrollment rate, a significant benefit has been observed with the employment of dedicated medical resources to screen patients, leading to an increase in enrollment. However, this possibility is limited by the availability of human resources [6]. Manual review of patient records for clinical trial eligibility represents a significant burden on research teams and medical oncologists. The manual extraction of this information is time consuming, labor-intensive, expensive, and error prone.

The information contained in electronic medical records is heterogeneous and complex. Extracting meaningful information from medical records presents significant challenges owing to the unstructured nature of these data, which limits their immediate applicability for clinical research [7]. As with other malignancies, information about patients with pancreatic cancer is primarily available in free-text form, which requires sophisticated processing. Rule-based extraction techniques are limited by their rigidity and the need for postprocessing. Machine learning and deep learning techniques show promise for recognizing word relationships; however, they require postprocessing for complex data and do not understand implicit or semantic information [8].

To address these limitations and challenges, we propose the use of a strategy for medical text mining with the help of large language models (LLMs) to convert text into structured computerized data using informatic extraction technologies. LLMs are high-performance technologies for classifying, filtering, and clustering information, enabling the direct extraction of information without additional postprocessing. These technologies can extract textual data from medical records and reorganize them for use in oncology research [9,10]. These technologies could also improve access to clinical trials in secondary centers or pediatric settings, with the expectation of improving health care democracy [11].

In PANCR-AI (Pancreatic Cancer Retrospective Screening with Artificial Intelligence) study, we assessed the performance of 3 different LLMs (GPT-4.5, OpenAI; Claude 3.7 Sonnet, Anthropic; and Mistral-7b-Instruct v0.3, Mistral) for clinical trial screening using a retrospective cohort of patients with metastatic or locally advanced pancreatic cancer [12-14].

## Methods

### Study Context and Population

The Institut de Cancérologie de Lorraine Comprehensive Cancer Center prospectively maintains a database of pancreatic malignancies. This database was queried to identify patients with newly diagnosed locally advanced, unresectable, or metastatic pancreatic adenocarcinoma. All patients with a pretherapeutic tumor board meeting report available in their electronic medical records between January 2018 and December 2023 were included in the study. Patients with other subtypes of pancreatic malignancies, such as intraductal papillary mucinous neoplasms or neuroendocrine tumors, were excluded. Each patient had a pretherapeutic tumor board meeting report summarizing their clinical and pathological status. For each patient, “candidate trials” were defined as all clinical trials open for enrollment in advanced or metastatic pancreatic cancer at the time of the tumor board meeting. Each possible match between a patient and a candidate trial constituted a patient-trial pairing, representing one evaluation of eligibility according to the trial’s inclusion and exclusion criteria. Clinical and pathological variables were extracted from the database and analyzed, including demographic characteristics, tumor pathology features, treatments received, and follow-up information. The study was registered with the French Health Data Hub on February 3, 2025 (22286214).

### Study Design and Data Source

This retrospective single-center analysis was based on pretherapeutic tumor board meeting reports, which provided a structured summary of each patient’s clinical condition and characteristics as documented in their medical records. These reports served as a common database for both oncologists and LLMs.

The inclusion and exclusion criteria were extracted from the sponsor’s trial protocol and reformatted into standardized textual items for analysis. Criteria related to biological samples, life expectancy, contraceptive use, or ethical consent were removed from the screening process due to the absence of corresponding data in the pretherapeutic tumor board meeting reports. The remaining criteria were independently reviewed and categorized according to their type (eg, clinical, pathological, or demographic) and analyzed for their textual characteristics, including word and character counts. The number of inclusion criteria ranged from 1 to 7, and the number of exclusion criteria ranged from 2 to 19. The characteristics of the 12 clinical trials are summarized in Table 1. Each criterion was evaluated for its number of characters, nature, and category of variables.

**Table 1.** Characteristics of the 12 candidate clinical trials open for recruitment (January 2018 to December 2023) and associated patient-trial pairs (N=341).

Clinical trials	ClinicalTrials.gov registry number	Phase	Sponsor type	Patients historically included in the trial (N)	Candidate patients for the trial (N)	Inclusion criteria analyzed, n (%)	Exclusion criteria analyzed, n (%)
Actuate 1801 - PDAC	NCT03678883	II	Industry	0	2	7 (58)	9 (75)
ALIX	NCT03974854	II	Health center	1	40	4 (57)	16 (84)
APACaP D-13	NCT02184663	N/A^a^	Medical association	1	43	5 (56)	6 (75)
AVENGERS 500 (PANC003)	NCT03504423	III	Industry	9	22	6 (50)	19 (76)
MAZEPPA GERCOR D19-02 PRODIGE-72	NCT04348045	II	Medical association	1	12	6 (35)	14 (88)
ONCOSNIPE PANCREAS	NCT04548960	II	Industry	0	12	1 (100)	2 (100)
OPTIMIZE-01	NCT04888312	Ib/II	Industry	3	21	6 (46)	10 (53)
PANDAS PRODIGE-44	NCT02676349	II	Health center	1	38	5 (42)	7 (78)
STEMNESS-PANC	NCT03721744	II/III	Industry	4	19	7 (47)	12 (92)
TEDOPaM study D17-01 - PRODIGE 63	NCT03806309	II	Medical association	7	55	4 (31)	20 (95)
URGENCE PANCREAS	NCT02979483	N/A	Medical association	5	44	3 (38)	3 (100)
EPIC	NCT02853474	III	Health center	0	33	5 (63)	2 (50)

a N/A: not applicable.

The inclusion and exclusion criteria for each candidate trial were evaluated on a criterion-by-criterion basis using information extracted from the pretherapeutic tumor board meeting reports. For each patient-trial pairing, fulfillment of every inclusion and exclusion criterion was assessed independently by the human gold standard and by each LLM. When the available clinical information in the reports was insufficient to assess a given criterion, or when it was not possible to reach a conclusion due to insufficiently consistent or contradictory information based on the tumor board meeting report, that criterion was considered to be met if it was an inclusion criterion and to be unmet if it was an exclusion criterion. This conservative assumption ensured that such patients were classified as eligible, reflecting a sensitivity-oriented approach consistent with the intended practical use of LLM-derived outputs. At the patient-trial level, the individual criterion assessments were then aggregated to determine the overall eligibility decision for each pairing.

### Performance Evaluation of LLMs

Three LLMs (ie, GPT-4.5, Claude 3.7 Sonnet, and Mistral-7B-Instruct v0.3) were evaluated for their ability to assess patient eligibility for each inclusion and exclusion criterion of all candidate trials based on the information contained in the pretherapeutic tumor board meeting reports. LLMs were queried through the online interfaces and respective application programming interfaces of OpenAI, Anthropic, and Mistral. The LLMs’ unique prompt design was developed according to European Medicines Agency guidelines and contextual prompt engineering methodology (role assignment, step-by-step reasoning, and constrained outputs) to ensure the reproducibility of the output for the same input. The input prompt is available in Multimedia Appendix 1 [15,16]. All LLMs were given the same instructions and data to be analyzed as the medical oncologists (for the human gold standard). Results are displayed in a dedicated radar plot. The screening time for each LLM was determined between the time each tumor board meeting was submitted and the time of completion of the output for each trial-patient assessment, measured using digital tools.

### Gold Standard Assessment

AC, AS, AM, LD, and CL are medical oncologists who participated in establishing the gold standard and volunteered to collect the data. Participation was proposed to a panel of physicians who were not involved in the care of the patients whose records were analyzed in this study to minimize potential bias in their assessments. The information provided for analysis included the inclusion and exclusion criteria for clinical trials and the deidentified tumor board meeting reports. The data were collected in a deidentified database stored on a secure internal server at the Comprehensive Cancer Center. All reports were reviewed by 2 medical oncologists to ensure reproducibility of the human assessment. Each oncologist was blinded to the evaluation of the other. In case of disagreement, a third medical oncologist independently reviewed the case to reach a consensus. Interrater reliability was assessed using Cohen κ coefficient. The screening time was reported by each medical oncologist for the determination of the gold standard and measured using digital tools. This duration was compiled and allocated to each corresponding candidate trial.

### Statistical Analysis

Data analyses were performed using SAS (version 9.4; SAS Institute Inc). *P*<.05 was considered statistically significant. Given the exploratory nature of the study, no adjustment for multiple testing was applied.

Quantitative data are reported as median values unless otherwise specified. Comparisons between 2 groups were performed using the Mann-Whitney test, and comparisons among more than 2 groups were performed using the Kruskal-Wallis test. Categorical variables were compared using the chi-square test. Survival was estimated using the Kaplan-Meier method, and survival curves were compared with the log-rank test.

For each inclusion and exclusion criterion, and subsequently at the patient-trial level, the LLM outputs were compared with the human gold standard. The results were classified as true positives when both methods identified eligibility; true negatives when both identified noneligibility; false positives when the LLM indicated eligibility, whereas the human gold standard did not; and false negatives when the LLM indicated noneligibility, whereas the human gold standard indicated eligibility. Cases in which the LLM and the human reviewers provided divergent results were considered as discordant.

LLM performance was evaluated using standard metrics (eg, recall, sensitivity, specificity, precision, and *F*_1_-score) to assess their ability to determine patient eligibility both at the level of individual inclusion criteria and across all clinical trials screened; sensitivity and specificity were computed with 95% CIs. Pairwise comparisons of LLM sensitivities (or specificities) were performed using McNemar test among cases identified as eligible (or ineligible) by the human gold standard.

Statistical analyses were conducted at the criterion level to assess factors associated with LLM-human discordance. Variables with *P*<0.1 in bivariate analyses were included in the multivariate logistic regression model.

### Ethical Considerations

The PANCR-AI study was conducted at the Institut de Cancérologie de Lorraine (Vandœuvre-lès-Nancy, France). It complies with the French reference methodology MR-004 of the French Data Protection Authority (Commission Nationale de l’Informatique et des Libertés) for research involving human data. According to the MR-004 regulatory framework, ethics committee approval and individual informed consent were not required, as the study involved retrospective analyses of deidentified clinical data and had no impact on patient care or management. For trial involvement, patients were evaluated using the standard manual screening process at the Institut Cancérologie de Lorraine. The reports were deidentified by retaining only age and gender, modifying dates to preserve the temporal relationship with the tumor board meeting, and implementing measures to ensure patient anonymity. The physicians who volunteered to collect the data were medical oncologists practicing at a comprehensive cancer center, and thus consent was implied.

## Results

### Study Population

Among the 141 patients initially identified from the Institut de Cancérologie de Lorraine Comprehensive Cancer Center database, 85 (60%) patients with locally advanced or metastatic pancreatic adenocarcinoma were included in this analysis; 46 (32.6%) patients were excluded due to unavailable pretherapeutic data in the tumor board meeting report, and 10 (7%) patients were excluded from the analysis based on histological type (all neuroendocrine tumors). The flowchart of eligibility is available in Multimedia Appendix 2. Overall, 30 (35.2%) patients of this cohort were historically included in a clinical trial. The median age was 68 (IQR 32‐89) years, and 49% (42/85) of patients were male. At the time of the tumor board meeting, 66 (78%) patients presented with metastatic disease, while 19 (22%) patients had locally advanced unresectable tumors. Patients were more likely to have been included in a clinical trial if the number of trials open for enrollment was higher at the time of the tumor board meeting (*P*=.02). Overall survival did not differ according to patients’ participation in clinical trials (*P*=.34). No significant association was observed between clinical trial inclusion and age (*P*=.09) or personal history of previous malignancy (*P*=.25). The baseline characteristics of the study population are summarized in Table 2.

**Table 2.** Characteristics of patients from the cohort of locally advanced and metastatic pancreatic cancer, according to inclusion or noninclusion in a clinical trial (N=341).

Characteristics, baseline, and demographics	All patients (n=85)	Patient historically included in the clinical trial (n=30)	Patient not included in the clinical trial (n=55)	*P* value
Age (years), median (IQR)	68 (60‐73)	62 (56‐67.25)	70 (61.25‐77.5)	.09
Female, n (%)	43 (51)	16 (53)	27 (49)	.82
Overall survival (months), median (95% CI)	9 (7-15)	14 (8‐18)	7 (5-13)	.34
Locally advanced pancreatic cancer, n (%)	19 (22)	5 (16)	14 (25)	.49
Candidate trials per patient, median (IQR)	4 (3‐5)	5 (4-6)	4 (2.5‐5)	.02
ECOG^a^, median (IQR)	1 (1‐2)	1 (1‐2)	1 (1‐2)	.86
History of malignancies, n (%)	7 (0.08)	1 (0.03)	6 (0.11)	.25
Cardiovascular comorbidities, n (%)	40 (47)	16 (53)	24 (43)	.53
BMI at diagnosis (kg/m^2^), median (IQR)	23.53 (20.96‐25.88)	24.01 (20.53‐27.12)	23.20 (21.36‐25.29)	.58

a ECOG: Eastern Cooperative Oncology Group Performance Status Scale.

### Gold Standard Assessment

Manual assessment of eligibility was performed as the gold standard for this analysis. Interrater reliability for manual assignment was substantial, with a Cohen κ value of 0.83 between reviewers (95% CI 0.81-0.84). Seven (0.06%) discordant cases required the intervention of a third reviewer to reach consensus.

### Performance of LLMs

**Figure 1.**
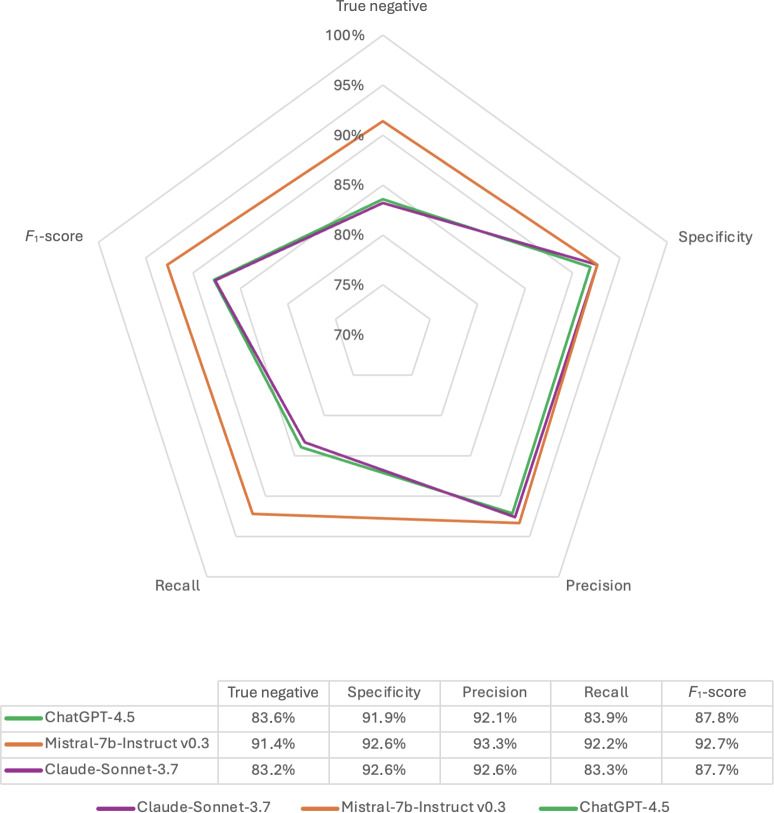
Radar plot of large language model performance compared to the human gold standard.

With a median of 4 (IQR 3-5) candidate trials per patient among the 85 patients analyzed, a total of 341 patient-trial pairs were identified. The overall performance of the 3 LLMs in evaluating patient eligibility for clinical trials is presented in Table 3. Detailed performance results of all LLMs for each trial are provided in Multimedia Appendix 3. Mistral-7b-Instruct v0.3 demonstrated the highest sensitivity, followed by GPT-4.5 and Claude 3.7 Sonnet (92.2%, 83.9%, and 83.3%, respectively), with a statistically significant difference between the Mistral-7b-Instruct v0.3 and the other 2 models (*P*<.01 for both comparisons). Overall performance metrics for each LLM are reported in Figure 1.

In clinical trials, analysis of the criteria revealed that the frequency of discordance between LLMs and human reviewers increased with the number of words and the number of characters in the criteria (*P*=.02 and *P*<.01, respectively). Both associations remained significant in a multivariable logistic regression model. The other characteristics of the criteria were not significantly associated with the risk of LLM error.

The total screening time for the gold standard manual assessment was 2682 minutes (ie, 44.70 hours). The total screening time using LLMs ranged from 152 minutes (2.53 hours) for Mistral-7B-Instruct v0.3 to 189 minutes (3.15 hours) for Claude 3.7 Sonnet. Screening time was significantly shorter for all LLMs compared to the gold standard assessment. Detailed screening time data are reported in Multimedia Appendix 4.

**Table 3.** Overall performance of large language models in selecting patients for clinical trials compared to the human gold standard based on the patient-trial pairs (N=341).

All	Number of true positive pairs	Number of false negative pairs	Number of true negative pairs	Number of false positive pairs	Sensitivity (95% CI)	Specificity (95% CI)
GPT-4.5	151	13	148	29	83.9% (78.5%‐89.3%)	91.9% (87.7%‐96.1%)
Claude 3.7 Sonnet	150	12	149	30	83.3% (77.9%‐88.8%)	92.6% (88.5%‐96.6%)
Mistral-7b-Instruct v0.3	166	12	149	14	92.2%^a^ (88.3%‐96.1%)	92.6% (88.5%‐96.6%)

a Statistically higher sensitivity.

## Discussion

### Main Findings

This study shows promising performance of LLMs for the prescreening of patients with locally advanced or metastatic pancreatic cancer based on pretherapeutic tumor board meeting reports. Screening for clinical trials assisted by LLMs would be appropriate in cases where there are a large number of inclusion and exclusion criteria or in peripheral centers with a view to referring patients to expert centers for a specific condition, thereby offering them the best chance of benefiting from therapeutic innovation.

The disparities in results between clinical trials using LLMs are difficult to interpret because of the small number of patients analyzed per trial, but overall, the risk of LLM error was correlated with the number of words or characters contained in a single inclusion or exclusion criterion. LLMs displayed poor performance in interpreting data, such as identifying clinically relevant past medical events or recent but resolved medical conditions. This aspect represents a major area for improvement in these technologies. No errors were observed in terms of timing between the discussion of the case in tumor board meetings and an event of particular interest. The identification of characteristics such as age, personal (rather than family) history of cancer, pregnancy status, or metastasis location was not a major cause of failure for LLMs. This is reassuring regarding the feasibility of using LLMs for the purpose of prescreening patients for clinical trials.

Mistral-7b-Instruct v0.3 is an LLM trained using multiple French-language databases and does not require translation when generating responses in French, unlike Anglo-Saxon models. Its optimization for Latin languages enables it to understand complex syntactic structures and semantic nuances. This characteristic may help explain the superior results obtained by this LLM, which outperformed the other 2 solutions tested.

### Comparison to Prior Work

Recent developments in AI and the rise of LLMs have offered promising opportunities to address these challenges. The ability of these models to process large quantities of complex patient data quickly and reliably has already demonstrated benefits in the field of oncology research [17]. Studies have shown that AI-based approaches significantly reduce the time required for screening eligibility, without compromising the consistency of trial-patient identification [18,19]. By integrating AI into clinical workflows, physicians could spend more time on direct patient interactions, complex medical decision-making, and personalized care, thereby enhancing overall health care quality and potentially improving patient outcomes [20,21]. Our study concurs with previous research, demonstrating the clear relevance of these tools in this setting [22]. A recent review focusing on the application of AI to clinical trial recruitment demonstrated several positive outcomes, including increased efficiency and improved recruitment or accuracy. It also emphasized various technical and ethical issues, including privacy, data security, transparency, and selection bias [23]. The first recent multicenter study using AI to streamline screening processes in patient medical records, conducted on a cohort of patients with lung cancer, found that LLMs achieved promising performance across 28 points of interest, including metastatic status and comorbidities. This study also highlighted that LLMs trained for screening make fewer errors than humans, although the contribution of humans to complement automated screening in cases where LLMs returned results with lower levels of confidence reduced the risk of error. The use of AI would also reduce variability between centers in identifying key features for clinical trial inclusion [24].

### Strengths and Limitations

Although the reasoning capabilities of generative AI models for clinical trial screening have shown impressive results, their implementation in clinical practice remains a challenge. The heterogeneity of software, data formats, extraction methods, and ethical considerations poses challenges to the routine use of these tools by health care providers.

Furthermore, because the analysis is based on a snapshot of the patient’s disease history, it is possible that some information may evolve over time, leading to misjudgment of certain inclusion or exclusion criteria for clinical trial screening. A significant number of tumor board meeting reports were missing due to the recent digitization of patient records. This study was also unable to use certain scanned documents of poor quality.

Given the limited number of patients and clinical trials, as well as the retrospective and single-center design of this study, the results should be interpreted with caution. However, they suggest a potentially valuable contribution of generative AI tools in screening patients with locally advanced or metastatic pancreatic cancer for their eligibility for clinical trials.

### Future Directions

Refining prompt engineering and fine-tuning models with domain-specific datasets could further enhance the accuracy and specificity of LLMs. Future work should explore integration with structured clinical data, such as laboratory values or radiological findings, to improve multimodal comprehension. Expanding the evaluation to a broader range of tumor types and multicenter datasets would improve generalizability.

The next step is to ensure outputs with clinical-grade standard requirements. Establishing a robust data governance framework to safeguard patient privacy, ensure data security, and maintain traceability of LLM actions could help to enhance acceptance of LLMs by regulatory agencies for real-time screening within electronic health records systems.

Finally, real-time prospective validation and workflow integration with electronic health records will be critical to assess the feasibility and clinical impact of LLM-assisted screening in daily oncology practice. Addressing these challenges will be essential to move from proof of concept to scalable and ethically sound clinical implementation.

## Supplementary material

10.2196/80268 Multimedia Appendix 1 Prompt used as a framework for analyzing clinical trial criteria for patients by both the human gold standard and large language models.

10.2196/80268 Multimedia Appendix 2 Flowchart of patient eligibility.

10.2196/80268 Multimedia Appendix 3 Overall and detailed performance of large language models for screening patients for each candidate trial.

10.2196/80268 Multimedia Appendix 4 Screening time per clinical trial (minutes).

## Abbreviations

AI: artificial intelligence
LLM: large language model
PANCR-AI: Pancreatic Cancer Retrospective Screening with Artificial Intelligence
