# Supplementary material for: AI-Driven Patient Screening for Clinical Trials in Pancreatic Cancer: The PANCR-AI Pilot Retrospective Comparative Study
Source: JMIR Cancer. 2026 Feb 23;12:e80268. doi: 10.2196/80268 (PMC12928684; doi:10.2196/80268)
Supplement: Multimedia Appendix 1 [file cancer-v12-e80268-s001.pdf]

Multimedia Appendix 1: prompt used as a framework for analyzing clinical trial criteria for patient by both human gold standard and LLMs.

STRUCTURED ANALYSIS METHOD

PURPOSE

To analyze a patient file with pancreatic cancer potentially with secondary metastatic lesions, with a view to inclusion in a clinical trial. Each inclusion and exclusion criterion were checked independently.

FUNDAMENTAL RULES

Treat each criterion independently as if no other information had been read beforehand.

Carry out a complete and rigorous double reading of the file for each criterion each time.

Do not assume or extrapolate the information. The information is strictly limited to that mentioned in the file. If any information is missing or incomplete, the criterion is coded 9.

ANALYSIS PROCEDURE

For each criterion:

1. Read the entire file to locate the necessary information.
2. Check the figures and dates: age, weight, indices, scores, and dates.
3. Check timeframes (e.g., history of cancer within 2 years).

REPORTING RESULTS MEANING

- 1: The situation described by the criterion is present = criterion met
- 0: The situation described by the criterion is absent = criterion not met
- 9: Impossible to conclude: data missing, incomplete, in progress, or subjective assessment

RESULT FORMAT TABLE

The table is generated exclusively from the codes indicated in the conclusions for each criterion x analyzed, without any further modification or interpretation.

One row per patient (file number)

One column per criterion, named: "inclusion x" or "exclusion x"

The coded value (1, 0 or 9) in each cell corresponds to criterion x.
