# Supplementary figures and images for: AI-Driven Patient Screening for Clinical Trials in Pancreatic Cancer: The PANCR-AI Pilot Retrospective Comparative Study
Source: JMIR Cancer. 2026 Feb 23;12:e80268. doi: 10.2196/80268 (PMC12928684; doi:10.2196/80268)

## Multimedia Appendix 2: Flowchart of eligibility of patient

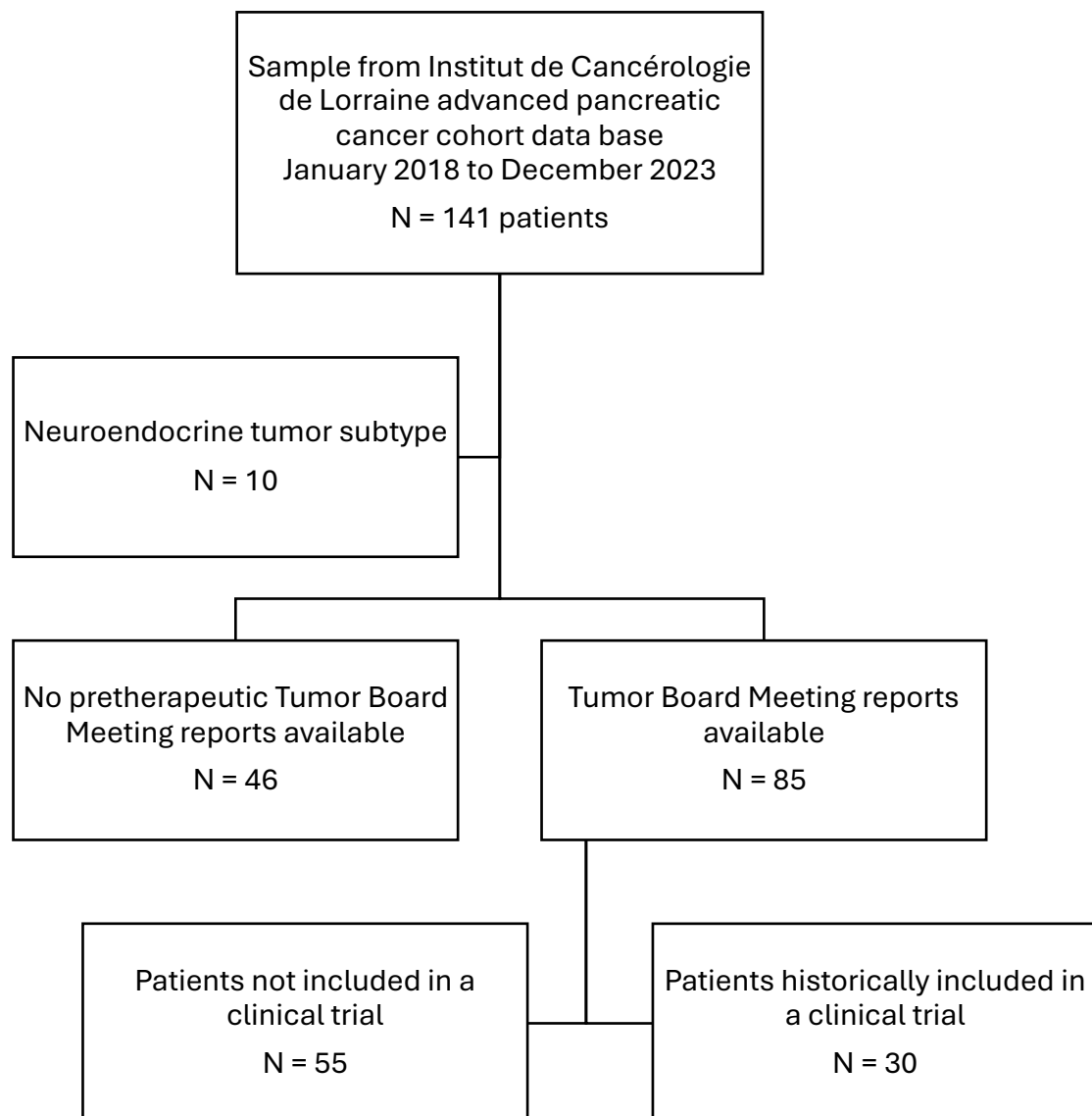

Supplement: Multimedia Appendix 2 [file cancer-v12-e80268-s002.pdf]
