# Supplementary material for: AI-Driven Patient Screening for Clinical Trials in Pancreatic Cancer: The PANCR-AI Pilot Retrospective Comparative Study
Source: JMIR Cancer. 2026 Feb 23;12:e80268. doi: 10.2196/80268 (PMC12928684; doi:10.2196/80268)
Supplement: Multimedia Appendix 3 [file cancer-v12-e80268-s003.pdf]

Multimedia Appendix 3: Overall and detailed performances of LLMs for screening patient per candidate trial.

All (N=341)	True Positive	False Negative	True Negative	False Positive	Sensitivity	Specificity
ChatGPT-4.5	151	13	148	29	83.9% [78.5%-89.3%]	91.9% [87.7%-96.1%]
Claude-3.7-Sonnet	150	12	149	30	83.3% [77.9%-88.8%]	92.6% [88.5%-96.6%]
Mistral-7b-Instruct v0.3	166	12	149	14	92.2% [88.3%-96.1%]	92.6% [88.5%-96.6%]
**TEDOPaM study D17-01 - PRODIGE 63 (N= 55)**						
ChatGPT-4.5	43	1	8	3	93.5% [86.3%-100.0%]	88.9% [68.4%-100.0%]
Claude-3.7-Sonnet	41	2	7	5	89.1% [80.1%-98.1%]	77.8% [50.6%-100.0%]
Mistral-7b-Instruct v0.3	46	2	7	0	100.0%	77.8% [50.6%-100.0%]
**URGENCE PANCREAS (N = 44)**						
ChatGPT-4.5	4	1	36	3	57.1% [20.5%-93.8%]	97.3% [92.1%-100.0%]
Claude-3.7-Sonnet	6	0	37	1	85.7% [59.8%-100.0%]	100.0%
Mistral-7b-Instruct v0.3	5	0	37	2	71.4% [38.0%-100.0%]	100.0%
**APACaP D-13 (N = 43)**						
ChatGPT-4.5	35	0	6	2	94.6% [87.3%-100.0%]	100.0%
Claude-3.7-Sonnet	34	2	4	3	91.9% [83.1%-100.0%]	66.7% [29.0%-100.0%]
Mistral-7b-Instruct v0.3	34	2	4	3	91.9% [83.1%-100.0%]	66.7% [29.0%-100.0%]
**ALIX (N = 40)**						
ChatGPT-4.5	4	4	31	1	80.0% [44.9%-100.0%]	88.6% [78.0%-99.1%]
Claude-3.7-Sonnet	4	2	33	1	80.0% [44.9%-100.0%]	94.3% [86.6%-100.0%]
Mistral-7b-Instruct v0.3	4	0	35	1	80.0% [44.9%-100.0%]	100.0%
**PANDAS PRODIGE-44 (N = 38)**						
ChatGPT-4.5	1	1	36	0	100.0%	97.3% [92.1%-100.0%]
Claude-3.7-Sonnet	1	3	34	0	100.0%	91.9% [83.1%-100.0%]
Mistral-7b-Instruct v0.3	1	0	37	0	100.0%	100.0%
**EPIC (N = 33)**						
ChatGPT-4.5	26	0	5	2	92.9% [83.3%-100.0%]	100.0%
Claude-3.7-Sonnet	24	1	4	4	85.7% [72.8%-98.7%]	80.0% [44.9%-100.0%]
Mistral-7b-Instruct v0.3	23	1	4	5	82.1% [68.0%-96.3%]	80.0% [44.9%-100.0%]
**AVENGERS 500 (PANC003) (N = 22)**						
ChatGPT-4.5	11	2	8	1	91.7% [76.0%-100.0%]	80.0% [55.2%-100.0%]
Claude-3.7-Sonnet	10	0	10	2	83.3% [62.3%-100.0%]	100.0%
Mistral-7b-Instruct v0.3	12	3	7	0	100.0%	70.0% [41.6%-98.4%]
**OPTIMIZE-01 (N = 21)**						
ChatGPT-4.5	9	1	9	2	81.8% [59.0%-100.0%]	90.0% [71.4%-100.0%]
Claude-3.7-Sonnet	9	2	8	2	81.8% [59.0%-100.0%]	80.0% [55.2%-100.0%]
Mistral-7b-Instruct v0.3	11	3	7	0	100.0%	70.0% [41.6%-98.4%]
**STEMNESS-PANC (N = 19)**						
ChatGPT-4.5	0	1	6	12	0.0% [0.0%-0.0%]	85.7% [59.8%-100.0%]
Claude-3.7-Sonnet	1	0	7	11	8.3% [0.0%-24.0%]	100.0%
Mistral-7b-Instruct v0.3	9	1	6	3	75.0% [50.5%-99.5%]	85.7% [59.8%-100.0%]

All (N=341)	True Positive	False Negative	True Negative	False Positive	Sensitivity	Specificity
**MAZEPPA GERCOR D19-02 PRODIGE-72 (N = 12)**						
ChatGPT-4.5	7	0	2	3	70.0% [41.6%-98.4%]	100.0%
Claude-3.7-Sonnet	10	0	2	0	100.0%	100.0%
Mistral-7b-Instruct v0.3	10	0	2	0	100.0%	100.0%
**ONCOSNIPE PANCREAS (N = 12)**						
ChatGPT-4.5	9	2	1	0	100.0%	33.3% [0.0%-86.7%]
Claude-3.7-Sonnet	9	0	3	0	100.0%	100.0%
Mistral-7b-Instruct v0.3	9	0	3	0	100.0%	100.0%
**ACTUATE 1801 – PDAC (N = 2)**						
ChatGPT-4.5	2	0	0	0	100.0%	NA
Claude-3.7-Sonnet	1	1	0	0	100.0%	0.0%
Mistral-7b-Instruct v0.3	2	0	0	0	100.0%	NA [0.0%-0.0%]
