# Supplementary material for: AI-Driven Patient Screening for Clinical Trials in Pancreatic Cancer: The PANCR-AI Pilot Retrospective Comparative Study
Source: JMIR Cancer. 2026 Feb 23;12:e80268. doi: 10.2196/80268 (PMC12928684; doi:10.2196/80268)
Supplement: Multimedia Appendix 4 [file cancer-v12-e80268-s004.pdf]

Multimedia Appendix 4: Screening time per clinical trial (minutes)

**Clinical trials**	**Gold standard assessment**	**ChatGPT-4.5**	**Claude-3.7-Sonnet**	**Mistral-7b-Instruct v0.3**	**p-value**
Actuate 1801 - PDAC	36	< 1	< 1	< 1	0.26
ALIX	330	17	21	19	<0.01
APACaP D-13	268	18	19	14	<0.01
AVENGERS 500 (PANC003)	168	7	11	13	<0.01
MAZEPPA GERCOR D19-02 PRODIGE-72	124	5	6	5	<0.01
ONCOSNIPE PANCREAS	64	2	3	2	<0.01
OPTIMIZE-01	316	12	14	10	<0.01
PANDAS PRODIGE-44	254	15	20	18	<0.01
STEMNESS-PANC	258	9	12	10	<0.01
TEDOPaM study D17-01 - PRODIGE 63	454	47	56	43	<0.01
URGENCE PANCREAS	186	7	9	5	<0.01
EPIC	224	15	18	12	<0.01
**Overall – (hours)**	2682 (44.70)	153 (2.55)	189 (3.14)	152 (2.53)	<0.01
